# Murine Typhus, Reunion, France, 2011–2013

**DOI:** 10.3201/eid2102.140850

**Published:** 2015-02

**Authors:** Elsa Balleydier, Guillaume Camuset, Cristina Socolovschi, Marie-Pierre Moiton, Barbara Kuli, Aurélie Foucher, Patrice Poubeau, Gianandrea Borgherini, Guillaume Wartel, Héla Audin, Didier Raoult, Laurent Filleul, Philippe Parola, Fréderic Pagès

**Affiliations:** Institut de Veille Sanitaire, Saint Denis, Réunion, France (E. Balleydier, L. Filleul, F. Pagès);; Centre Hospitalier Universitaire Réunion, Saint Pierre, Réunion (G. Camuset, A. Foucher, P. Poubeau, G. Borgherini, G. Wartel);; Unité de Recherche sur les Maladies Infectieuses et Tropicales Émergentes, Marseille, France (C. Socolovschi, D. Raoult, P. Parola);; Centre Hospitalier Universitaire Réunion, Saint-Denis, Réunion (M.-P. Moiton, B. Kuli);; Centre Hospitalier Gabriel Martin, Saint-Paul, Réunion (H. Audin)

**Keywords:** Fleas, murine typhus, Reunion, rodents, bacteria, zoonoses, vector-borne infections, France

## Abstract

Murine typhus case was initially identified in Reunion, France, in 2012 in a tourist. Our investigation confirmed 8 autochthonous cases that occurred during January 2011–January 2013 in Reunion. Murine typhus should be considered in local patients and in travelers returning from Reunion who have fevers of unknown origin.

Murine typhus, an acute zoonotic infection caused by *Rickettsia typhi* (*1*), occurs worldwide. It is underdiagnosed and largely underreported because of its nonspecific characteristics and frequently mild course, a lack of active monitoring; and limited awareness among physicians (*2*). Symptoms include fever, headache, and inconsistent and transient rashes (*1*,*3*). Serious complications have been associated with acute infections (*4*). The death rate is generally low but can reach 4% without the use of antibacterial drugs (*1*,*4*). The classic cycle involves rats (black rat [*Rattus rattus*] and brown rat [*R. norvegicus*]) in urban areas and the rat flea *Xenopsylla cheopis* (*3*). However, murine typhus is also documented in suburban areas, where opossums, cats, dogs, and their fleas coexist (*3*).

Reunion is a French overseas territory of 2,512 km^2^ in the southwestern Indian Ocean, 700 km east of Madagascar, and has a tropical climate. Most of its 850,000 residents live in coastal areas where major towns are located. Invasive rodents (rats, including the black rat, mice, and shrews) and endemic rodents (i.e., the tailless tenrec [*Tenrec ecaudatus*]) are present both in rural and urban settings (*5*). *X. cheopis* and *X. brasiliensis* fleas are present mainly on rats, but their presence and their abundance vary according to the local climates of the island and to the seasons (*6*). Stray dogs and feral cats are numerous in the island and often are infested with fleas. In addition, domestic dogs and cats traditionally are allowed to run freely in both urban and rural areas.

In early 2012, murine typhus was reported in a tourist returning from Reunion (*7*). We conducted a study to collect epidemiologic data and to describe clinical manifestations for all murine typhus cases diagnosed in the infectious disease departments of hospitals in Reunion.

## The Study

In agreement with infectious disease practitioners and the World Health Organization (WHO) Collaborating Centre for Rickettsial Diseases, we chose to research murine typhus only in cases of fever of unknown origin lasting >7 days. In accordance with WHO Collaborating Centre procedures, a case was considered confirmed by seroconversion or by a >4-fold increase in antibody response against *R. typhi* from acute- and convalescent-phase serum samples; by a positive indirect immunofluorescence test for typhus-group rickettsiae confirmed by Western blot; or by positive *R. typhi*–specific real-time quantitative PCR. We used Western blot to formally distinguish murine typhus from epidemic typhus. Routine analysis (i.e., blood cell count) was performed in Reunion. Murine typhus diagnostic tests were performed by the WHO Collaborating Centre for Rickettsial Diseases (Marseille, France) (*7*).

We collected and analyzed data for each case by reviewing clinical records and administering an epidemiologic questionnaire by phone to each patient. The following data were collected: symptoms, signs, and complications during illness; results of routine laboratory analyses; treatment and outcome; habitat and environmental characteristics; presence of pets, livestock, or rodents; contact with fleas; daily activities in the 15 days before symptom onset; and travel during the previous 3 months.

**Figure 1:**
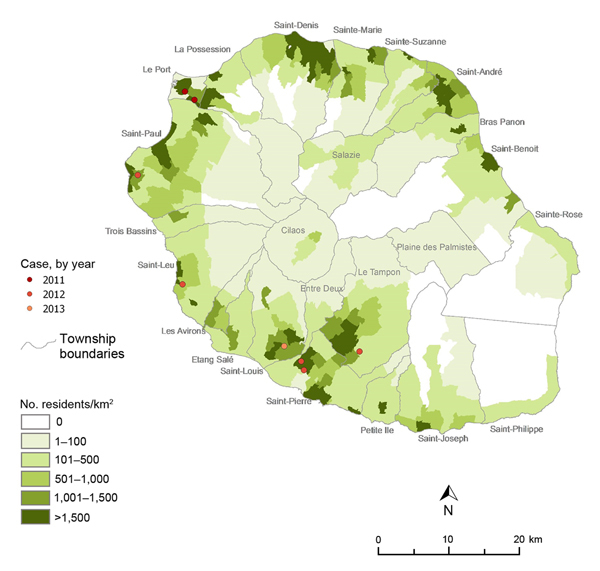
Locations of murine typhus cases, Reunion, France, January 2011–January 2013.

**Figure 2:**
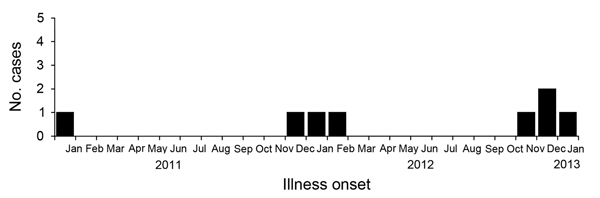
Temporal distribution of murine typhus cases, Reunion, France, January 2011–January 2013.

During January 2011–January 2013, a total of 8 autochthonous murine typhus cases were confirmed: 5 patients in 2012, 1 in 2013, and 2 who tested positive for retrospective screening of archived serum samples from 2011 (Figures 1, 2; Table). Seven cases occurred during the Southern Hemisphere summer. All the patients lived in private houses in the western and southern parts of the island in periurban areas. Patients’ average age was 46 years (range 21–66 years), and the male:female ratio was 1:1.

**Table:** Confirmation of 8 murine typhus cases, Reunion, France, January 2011–January 2013*

Pt no.	Age, y/sex	Onset date	Onset to first sample, d	First test result		First to second sample, d	Second test result		Second to third sample, d	Third test result			Confirmatory test results		Onset to PCR, d
				IgM†	IgG‡		IgM†	IgG‡		IgM†	IgG‡		WB	PCR	
1	55/M	2011 Jan	18	512	256	76	0	256					*R. typhi*	ND	
2	66/M	2011 Dec	30	512	128	63	128	128					*R. typhi*	ND	
3	41/F	2012 Jan	33	512	128	66	512	256					*R. typhi*	Neg	17
4	49/M	2012 Feb	14	0	0	22	512	128	39	128	128		*R. typhi*	Neg	14
5	47/F	2012 Nov	12	256	128	26	256	512					ND	ND	
6	50/M	2012 Dec	11	0	0	19	512	256					ND	Pos	11
7	21/F	2012 Dec	9	128	128	48	512	256					ND	ND	
8	39/F	2013 Jan	37	512	128	172	0	512					ND	ND	

*All samples were serum samples. Blank cells indicate a third serum sample was not tested. ND, not done; neg, negative; pos, positive; pt, patient; WB, Western blot. †IgM pos if titer >1:64. ‡IgG pos if titer >1:128.

For all patients, fever lasted an average of 14.3 days (range 10–21 days). Six patients were hospitalized. The 2 other main symptoms were arthromyalgia (7 patients) and headaches (6 patients). Only 4 patients had a maculopapular rash, but no inoculation eschar was found. In addition, pharyngitis developed in 4 patients. In contrast, gastrointestinal symptoms and ophthalmologic signs, such as uveitis, developed in 2 patients each. One patient had confusion, and 1 had prostration.

Six patients had elevated liver enzymes (reference values aspartate aminotransferase and alanine aminotransferase >33 UI/L; lactate dehydrogenase >480 UI/L). Early lymphopenia (lymphocytes <1,000 cells/mL) occurred in 5 patients, and thrombocytopenia (platelets <150 000/mL) in 4. Renal dysfunction did not develop in any of the 8 patients.

Six patients were treated with doxycycline (2 patients in the acute phase for whom fever decreased during the following 24 hours; 4 others despite previous spontaneous resolution of fever). One of the 2 remaining patients, a pregnant woman, was treated with spiramycin without any complications to the baby during 6 months of follow-up. The other patient did not receive any treatment. All patients recovered completely, although they had weakness lasting at least 1 month.

None of the patients had traveled overseas during the previous 3 months, and no other cases had been identified nearby. Only 1 patient reported having recent insect bites and pruritus. No patient recalled contact with rats in his/her living environment before disease onset.

Five patients completed the investigation questionnaire. Reported risk factors included close contact with domestic pets (4 patients); presence of livestock in the surroundings (4 patients); presence of wild fauna (rats, tenrecs, dogs, cats) in the surroundings (4 patients); rat extermination (3 patients); outdoor activities, such as jogging, picnicking, walking, gardening (3 patients); or house cleaning (2 patients).

## Conclusions

Serologic and molecular evidence is sufficient to indicate that autochthonous transmission of murine typhus exists in Reunion. The real impact and the origin (always present but only recently identified vs. recent importation by navigation trade from Asia or from Madagascar) of murine typhus remain unknown. Murine typhus is common in tropical areas but is often not distinguished from other more prevalent febrile bacterial infections, such as scrub typhus, spotted fever rickettsioses, and leptospirosis. However, murine typhus recently has been shown to be a major cause of fever of unknown origin in Indonesia and Laos (*8*,*9*). In Reunion, despite a systematic biologic investigation (dengue, chikungunya, and leptospirosis) of dengue-like syndrome, many fevers still remain of unknown origin (*10*,*11*). Our results support the hypothesis that murine typhus could be a major cause of fever in Reunion.

Like leptospirosis, murine typhus seems to peak seasonally during the summer (*12*). As previously described in the tropics, climatic conditions during the hot and wet seasons lead to the proliferation of rats and so increase human exposure to murine typhus (*4*,*13*). Because most of the 8 patients had contact with pets, the role of fleas that infest pets (e.g., *Ctenocephalides felis*) in the transmission of murine typhus needs to be clarified, as does the role of *X. cheopis*, the rat flea (*14*).

In conclusion, murine typhus is an emerging disease in Reunion. It is possibly underdiagnosed and has the potential to cause major illness (*9*). This disease should be considered locally when fevers of unknown origin are investigated, as well as in travelers with fever returning from the island. Specific studies are needed to assess the effect of murine typhus on public health and to describe the epidemiology of the disease. The best way to prevent murine typhus is to minimize exposure to ectoparasite vectors by limiting contact with rodents and fleas. Such measures include keeping a well-maintained yard, minimizing waste, protecting pets from fleas, and using mechanical or chemical protection in case of at-risk activities.
